# Soil depths and microhabitats shape soil and root-associated bacterial and archaeal communities more than crop rotation in wheat

**DOI:** 10.3389/frmbi.2024.1335791

**Published:** 2024-02-05

**Authors:** Adriana Giongo, Jessica Arnhold, Dennis Grunwald, Kornelia Smalla, Andrea Braun-Kiewnick

**Affiliations:** ^1^ Institute for Epidemiology and Pathogen Diagnostics, Julius Kühn-Institute, Federal Research Centre for Cultivated Plants, Braunschweig, Germany; ^2^ Institute of Sugar Beet Research, Göttingen, Germany

**Keywords:** continuous wheat, N-cycling functional genes, plant microbiome, rhizoplane, rhizosphere, root-associated soil, wheat monoculture

## Abstract

The plethora of microorganisms inhabiting the immediate vicinity of healthy root systems plays a pivotal role in facilitating optimal nutrient and water acquisition by plants. In this study, we investigated the soil microbial communities associated with wheat roots within distinct microhabitats, root-affected soil (RA), rhizosphere (RH), and rhizoplane (RP). These microhabitats were explored at five soil depths, and our investigation focused on wheat cultivated in a monoculture (WM) and wheat crop rotation (WR). Overall, there were significant differences in microbiota composition between WM and WR, although no difference in bacterial diversity was observed. Differentially abundant taxa between WM and WR were observed in all three microhabitats, emphasizing important insights on the localization of commonly associated bacteria to wheat roots. Comparing the microhabitats, RP exhibited the most dissimilar microbial composition between WM and WR. Taxa that were differentially abundant between WM and WR were observed in the three microhabitats. The high relative abundance of taxa belonging to the phylum *Proteobacteria* in the rhizoplane, such as *Devosia*, *Pseudomonas*, *Shinella*, and *Sphingomonas*, along with other genera, such as *Pedobacter* (*Bacteroidota*), *Agromyces* and *Streptomyces* (*Actinobacteriota*) highlight the recruitment of potentially beneficial bacterial taxa to the vicinity of the roots. Interestingly, these taxa were observed along the entire length of wheat roots, even at depths of up to 120 cm. The presence of specific taxa associated with wheat roots at all soil depths may be beneficial for coping with nutrient and water shortages, particularly under upcoming climate scenarios, where water may be a limiting factor for plant growth. This study provides valuable insights for designing management strategies to promote a diverse and healthy microbial community in wheat cropping systems, considering soil depth and microhabitats as key factors. Although, at this time, we cannot link specific bacterial taxa to yield reductions commonly observed in monocultural fields, we propose that some genera may enhance plant nutrient or water acquisition in rotation compared with monoculture. Advanced technologies, including functional analyses and culturomics, may further enhance our understanding of the ecological roles played by these microbes and their potential applications in sustainable agriculture.

## 1 Introduction

Healthy root systems facilitate essential functions such as nutrient absorption, water uptake, overall plant growth, and crop yield. The interaction between roots and microorganisms is a crucial aspect of this process. Intensive crop cultivation as a monoculture may lead to an imbalance in the soil microbiome and a decrease in plant health and productivity (Berg & Cernava, 2022). This model of crop cultivation might result in repeated exposure of the soil, crop, and microbiome to the same environmental drivers brought about by the crop, rising soil exhaustion, soil-borne diseases, and pests (Bennett et al., 2012; Peralta et al., 2018; Wu et al., 2020; Kavamura et al., 2021; Palma-Guerrero et al., 2021; Wang et al., 2021).

Root surfaces are critical sites for interactions between plants and their associated microorganisms. In conjunction with the roots, the associated soil forms microniches that exhibit complexity, resulting in distinct microhabitats. Extensive research has identified well-defined microhabitats, including bulk soil, rhizosphere, rhizoplane, and endosphere, characterized by specific bacterial diversity and composition (Xiong et al., 2021). The rhizosphere encompasses a collection of processes in the root-soil interface, known as the “rhizosphere effect” (Hiltner, 1904; Starkey, 1938). These processes include root exudation, microbial activity, genetic exchange, nutrient transformation, and gradient diffusion. Together with the rhizoplane, the specific area of the root surface in direct contact with the soil (Foster, 1986), which includes the epidermis and root mucilage, forms the area where the plant invests in rhizodeposition - the exudation of available and degradable organic carbon substrates for the rhizosphere - and interactions with microorganisms (Leyval & Berthelin, 1993). The plant is rewarded with increased nutrient and phosphorus availability through microbial mining, enzymatic soil organic matter mobilization, and fast-cycling N reservoir degradation (Bulgarelli et al., 2013; Hosseini et al., 2022). These conversion processes are primarily conducted by microorganisms nourished by root exudates (Meier et al., 2017), which host the enzymes responsible for facilitating nitrogen transformation processes, including diazotrophs, denitrifiers, and nitrifiers (Das et al., 2022; Liu et al., 2023). The rhizoplane also encompasses a communication portal between the plant and the microorganisms, supporting the transit of biomolecules, nutrient uptake, the availability of root exudates, controlling the entry of harmful microorganisms into the plant tissue, and microbiota-assisted priming against plant diseases (Edwards et al., 2015).

Deep subsoil microbiota close to the roots is crucial for agricultural soil system functioning (Ryan et al., 2023) and nutrient cycling (Beule et al., 2022; Naylor et al., 2022; Naylor et al., 2023), especially in agricultural production systems during drought, low nutrient supply, and extensive agriculture practices. Most root microbiome studies are conducted at the plow horizon (0-30 cm), where most roots are located (Maeght et al., 2013; Simonin et al., 2020; Jaiswal et al., 2022; Naylor et al., 2022). Winter wheat can grow up to 150 cm in depth (Fan et al., 2016), with an effective root zone estimated at ~ 80-100 cm. Thus, the root cover area is far deeper than the plow horizon investigated in most soil microbiome studies. The role of deep roots in plant development and food production and how they are affected by crop rotation should be better elucidated.

Crop rotations offer a sustainable alternative to monoculture by preserving soil fertility and increasing productivity (Leteinturier et al., 2006; Tiemann et al., 2015; Babin et al., 2019; Woo et al., 2022). Crop rotation also contributes significantly to biodiversity enhancement (Albrecht, 2003) and improvements in the soil microbiome (Liu et al., 2023). Prior research on various crop rotations has primarily examined the overall soil microbiome, often overlooking distinctions among plant microenvironments, such as rhizo-compartments or soil depths. To comprehend the dynamics of bacterial and archaeal (referred to as microbial hereafter) communities within the rhizosphere of winter wheat in the context of crop rotation and monoculture, our study focused on the impact of soil depth (layers) and soil microenvironments at increasing distances from the roots.

Our research firstly hypothesized that there are significant disparities between the microbial communities in rotational wheat and wheat cultivated in monoculture, providing insights into the impact of monoculture practices on crop ecosystems. Secondly, the soil depth influences the diversity and composition of the microbial community associated with the wheat roots differently in these two crop systems.

## 2 Material and methods

### 2.1 Experimental study site design

The field site is located northeast of Harste, Central Germany (51°36’23.5” N, 9°51’55.8” E), where the crop rotation trial was established in 2006 (Koch et al., 2018). Two winter wheat (*Triticum aestivum* L.) rotations were included in this study: a six-year crop rotation (WR) with winter oilseed rape – winter wheat – winter wheat – grain pea – sugar beet – winter wheat, of which the first and second wheat after oilseed rape were sampled in this study, and a 15-year wheat monoculture (WM). Each crop element was grown in the same area across sequential growing seasons. The plot size was 16.2 x 14.0 m, and each field plot was replicated three times in blocks per rotation. The soil type is a silty loam Luvisol derived from loess (IUSS Working Group WRB, 2015). Nitrogen fertilization was used to achieve an optimal N supply of 265 kg N ha^-1^ based on mineral nitrogen (N_min_) soil analysis in spring. The soil pH in the 0-30 cm soil depth was 6.8 in WM and 6.7 in WR.

### 2.2 Soil core sampling and processing

Eight vertical soil cores were sampled at the wheat developmental growth stage BBCH 69 at the same time (June 2020) using a specific drill pipe connected to a tractor to extract soil cores with a diameter of 6 cm and a length of 120 cm. After removing all above-ground plant parts, the drill pipe was positioned above the wheat plant. Each core was pushed out of the drill head into a plastic bag. Four cores were taken from WR plots (two from the first and two from the second wheat after oilseed rape) and four from WM plots (n = 4 cores for each treatment). All cores were placed on ice in the field and stored at 4 °C overnight before further microbiological analysis (**Supplementary Figure 1**).

Four core replicates of each treatment were split into five different soil layers (L1, 0-15 cm; L2, 15-30 cm; L3, 30-60 cm; L4, 60-90 cm; and L5, 90-120 cm depth) and three rhizo-compartments (therefore called microhabitats), resulting in 120 samples. Microbial communities from different microhabitats were obtained according to the method described by Lucas et al. (2018), with minor modifications. Briefly, loosely adhered root soil (root-affected soil, or RA) was obtained by vigorously shaking the total root mass collected from each soil core segment. After this procedure, the roots still had a layer of soil attached to them (rhizosphere, or RH). The complete root system was placed on a sterile surface, and the RH soil was gently brushed off the root surface with a sterile toothbrush. Root pieces from each sample were collected, weighed, and placed in a sterile 15-mL conical tube to collect the adhering soil/cells from the root surface (rhizoplane, or RP). A 1:10 volume of 0.3% NaCl was added according to the root weight (1 g of fresh root weight plus 9 mL of buffer), and the tube was vortexed at the highest speed for 1 min. The supernatant was transferred to new tubes without roots. The solution was centrifuged at 10,000 *g* for 30 min at 4°C. The pellets obtained (RP) were stored at -20°C until DNA extraction. The cleaned leftover roots were dried with a paper towel, and the fresh weight of the roots (FW) was determined.

### 2.3 Soil DNA extraction and sequencing

Total DNA was extracted from 0.5 g of RA and RH soil samples using the FastDNA SpinKit for Soil (MP Biomedicals, Santa Ana, USA) and a maximum of 0.5 g of roots in RP according to the manufacturer’s instructions, except that the DNA from RA and RH were eluted in 100 µL of DNase free water (DES), and RP samples were eluted in 80 µL. The concentration and integrity of the total DNA were checked by Nanodrop (Thermo Fisher, Waltham, USA) and on a 0.8% agarose gel, respectively.

DNA for amplicon sequencing was tested with a pre-Illumina PCR reaction using the V3-V4 region of the 16S rRNA gene primers Uni341F (5′ CCTAYGGGRBGCASCAG 3′) and Uni806R (5′ GGACTACHVGGGTWTCTAAT 3′) (Yu et al., 2005; Caporaso et al., 2011; Sundberg et al., 2013). PCR products were checked on a 1% agarose gel. The amplicon libraries were generated using primers Uni341F-Uni806R with Illumina adaptors (Illumina, San Diego, USA). They were sequenced at Novogene (UK) using Illumina MiSeq v.2 (2 x 250 bp) chemistry according to the manufacturer’s instructions. Unassembled raw amplicon data were deposited in the National Center for Biotechnology Information (NCBI) Sequence Read Archive (SRA) under BioProject PRJNA940322.

### 2.4 Quantification of bacterial 16S rRNA, *nifH*, *nosZ*, and *amoA* genes by qPCR

The quantification of bacterial 16S rRNA gene copies and N-cycle-related genes, including the nitrogenase gene (*nifH*), nitrous oxide reductase gene (*nosZ*), and ammonia monooxygenase alpha subunit gene (*amoA*) were conducted through quantitative PCR (qPCR) analysis in the RP samples. To quantify absolute bacterial abundance, the BACT1369F, PROK1492R, and TM1389F primers (with 5’-FAM and 3’-TAMRA labels) were employed in a TaqMan assay, following the protocol described by Suzuki et al. (2000). To estimate the abundance of the *nifH* gene, the FPGH19 primer (Simonet et al., 1991) was used along with the PolR primer (Poly et al., 2001). For quantifying the *nosZ* gene, the *nosZ2F* and *nosZ*2R primers were used (Henry et al., 2006). Finally, the bacterial *amoA* gene abundance was determined using the *amoA*-1F and *amoA*-2R primers (Rotthauwe et al., 1997). The specificity of the amplification products employing SYBR green chemistry (New England Biolabs, Ipswich, USA) was confirmed through melting curve analyses. The quantification of all target gene copies in the samples was determined by comparing them to adequate standard curves of cloned and purified target gene copies, and all measurements were based on 1 g of fresh roots. Standard curves were generated by serial dilutions of target genes, including part of the 16S rRNA gene from *E. coli*. Reference DNAs for bacterial *nifH*, *nosZ*, and *amoA* genes were used based on purified gene fragments inserted into either the pEASY-T1 or pCR2.1 cloning vectors and transformed into *E. coli*. All measurements were duplicated using a CFX96 Real-Time System (Bio-Rad, Hercules, USA).

To analyze the log-transformed gene copy number quantification data, we performed a three-way ANOVA followed by Tukey’s HSD test (*p* < 0.05). This analysis used the estimated marginal means implemented in the emmeans R package (Lenth et al., 2023). To assess the presence of the target N-cycle-related genes relative to the overall bacterial population, the gene proportions were calculated by comparing each specific gene of interest with the total count of bacterial 16S rRNA gene copies (Das et al., 2022).

### 2.5 Amplicon sequence analyses

Sequences were processed and classified using the Divisive Amplicon Denoising Algorithm (DADA2 v.1.12.1 pipeline; Callahan et al., 2016) in R version 4.1.3 (R Core Team, 2019). The quality trimming and filtering steps were performed using the “FilterAndTrimmed” function. Reads of less than 100 bp were removed, and two expected errors per read were allowed. After quality filtering, denoising, and chimera removal, 16S rRNA gene amplicon depths yielded 8,591,570 high-quality reads from 120 samples, corresponding to an average of 71,596 reads per sample (**Supplementary Table 1**).

The amplicon sequencing variants (ASVs) were taxonomically assigned based on the SILVA database v.138 (Quast et al., 2013) and imported into the phyloseq package (McMurdie & Holmes, 2013). ASVs unassigned at the phylum level and any residual ASVs identified as chloroplasts, mitochondria, or eukaryotes were excluded from the analyses. The phyla nomenclature was maintained as suggested by the SILVA database. *Thaumarchaeota*, an archaeal phylum that might require manual curation to align its genera with the updated classification (previously identified as the mesophilic *Crenarchaeota*; Brochier-Armanet et al., 2008), had both names displayed in the text. Amplicon sequencing resulted in 20,065 unique ASVs for WM and 17,947 for WR (**Supplementary Table 1**). The rarefaction curves tended to reach a saturation plateau, indicating that the sequencing approach provided sufficient sequences to cover most of the diversity in the samples.

Alpha diversity was computed for rarefied reads after examining the sequencing depth using the vegan package (Oksanen et al., 2020). Sequences were rarefied for the lowest number of sequences identified among the samples (minimum of 14,438 sequences). Using 10,000 permutations, Kruskal–Wallis tests were employed to detect statistically significant changes in alpha diversity. For categories in which the Kruskal–Wallis test led to the rejection of the null hypothesis (*p* < 0.05), a *post hoc* Wilcoxon–Mann–Whitney test was conducted.

Beta diversity was assessed using the square root transformed ASV count data. The Bray-Curtis dissimilarity index was calculated to obtain a distance matrix among the samples. Permutation analysis of variance (PERMANOVA) with 10,000 permutations was used to assess the statistical significance of these comparisons. Multidimensional scaling analysis (MDS, also known as Principal Coordinate Analysis, PCoA) was used to visualize the microbial communities in WM and WR. Differences in beta diversity centroids were assessed using permutational multivariate analysis of variance (*adonis* test) and PERMANOVA in the vegan package (Oksanen et al., 2020). Differences in beta diversity between microhabitats and layers by looking at the principal effect of each variable separately were assessed with an Analysis of Similarity (ANOSIM) on 10,000 permutations using the vegan package. The presence of every taxon (unique and shared) in a total or specific composition of samples was verified using the limma package (Ritchie et al., 2015) on absolute abundance data and visualized using the VennDiagram package (Chen, 2021a). The microbial composition of the samples was visualized after transformation to relative abundance. To identify taxa with significant differences across WM and WR, we used a negative binomial Wald test implemented in DESeq2 v1.18.1 within the phyloseq package to test for differential abundance (DA) on unrarefied reads (Love et al., 2014). After the Benjamini-Hochberg correction method, the taxa were considered differentially abundant when the adjusted *p*-value was below 0.05.

## 3 Results

### 3.1 Root biomass and quantification of the 16S rRNA, *nifH*, *nosZ*, and *amoA* genes along a depth gradient

**Figure 1:**
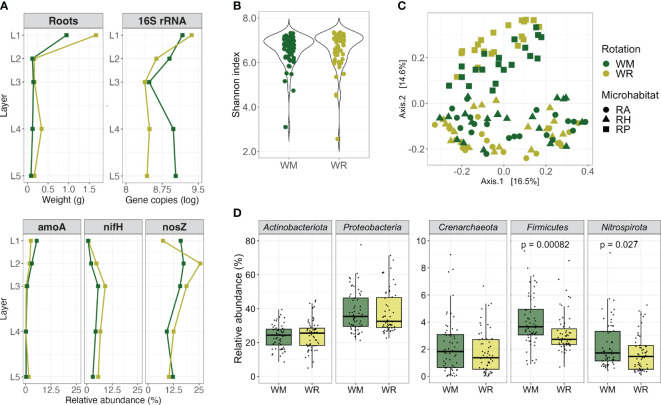
Comparison between crop rotational positions, wheat monoculture (WM), and wheat rotation (WR). **(A)** Root fresh weight, 16S rRNA gene copies per gram of root fresh weight, and relative abundance of N-cycle related genes by depth; **(B)** alpha diversity (Shannon index, within-group microbial diversity); **(C)** beta diversity measurement of dissimilarity between microbial communities and PERMANOVA analyses of main factors that might shape the wheat microbiota; and **(D)** the composition of the main microbial phyla. Groups sharing the same letter are not significantly different. Red asterisks indicate significant differences (*p* < 0.05) between the groups when comparing WM and WR.

The total fresh root biomass obtained from each segment of the core was significantly higher in the top layer, L1, than in the deeper layers in both WM and WR (*p* = 0.0087 and *p* = 0.00049, respectively). In L1, WR produced 2.4 times more fresh root biomass than WM (0.94 ± 0.29 g for WM and 2.3 ± 0.72 g for WR; *p* = 0.0629) (**Figure 1A**).

The absolute and relative abundance of specific target genes per gram of root by depth was measured by qPCR only in the RP since it was the microhabitat closest to the roots. Regarding 16S rRNA gene copy numbers, there was a consistent and significant decrease from L1 to the deeper soil layers, which agreed with the decreasing amount of root material (**Figure 1A**; WM, *p* = 0.0091 and WR, *p* = 0.00219). WR presented higher gene copy numbers in L1 than in L2 (*p* = 0.0082); there were no significant differences in the other layers. The lowest gene copy number in WM was noticed in L3, whereas it increased again in L4 and L5. Thus, when rotations were compared, a significantly higher 16S rRNA gene copy number was observed in WM than in WR (L4, *p* = 0.0496; L5, *p* = 0.0156).

The relative abundance of the *amoA* gene followed a trend, with its highest levels observed in the L1, averaging 5.5% of the 16S rRNA genes in WM and 2.6% in WR. However, as it penetrated deeper into the soil, its relative abundance dropped to less than 1% (**Figure 1A**). Conversely, the *nifH* gene relatively increased from the L1 to L3 and decreased in the deeper layers, following a consistent pattern for WM and WR. The *nosZ* gene relative abundance differed between WM and WR. While WM displayed approximately twice the abundance of *nosZ* in L1 compared with WR, *nosZ* increased in L2 in WR. Then, in the deeper layers, *nosZ* abundance in WM declined to a similar level observed in WR. Remarkably, even as we sampled deeper into the soil, *nosZ* maintained a significant relative abundance in WM and WR, reaching a higher relative abundance in L3 (18.8% of the 16S rRNA genes) but still with 12.2% and 10.2% in WM L5 and WR L5, respectively.

### 3.2 Microbial diversity and composition under wheat monoculture and crop rotation scenarios

The alpha diversity was similar in WM and WR when considering various microhabitats and depths (Wilcoxon test; Shannon, *p* = 0.16; Chao1, *p* = 0.20; Pielou, *p* = 0.17) (**Figure 1B**; **Supplementary Table 2**). However, when visualizing dissimilarities in microbial communities using multidimensional scaling (MDS) plots based on Bray-Curtis distances, significant distinctions between the WM and WR groups were evident (PERMANOVA, *p* < 0.001) (**Figure 1C**). These differences underscore the influence of crop rotation, microhabitats, and depth (layer) on microbial communities. The microhabitat emerged as the most influential factor, contributing to 15.5% of the observed variation, depth at 14.4%, and rotation at 4.9% (**Supplementary Table 3**).

The predominant bacterial phyla, *Actinobacteriota* and *Proteobacteria*, along with the dominant archaeal phylum, *Crenarchaeota* (*Thaumarchaeota*), displayed similar relative abundances in both WM and WR samples (*p* > 0.05) (**Figure 1D**) when all the samples were collectively analyzed. These phyla accounted for 24.1%, 38.3%, and 2.0% of the total sequences, respectively. Notably, differences between WM and WR were observed at the phylum level. *Firmicutes* and *Nitrospirota* were significantly more abundant in WM than in WR (*p* = 0.00082 and *p* = 0.027, respectively). In WM, *Firmicutes* accounted for an average of 4.2% of the total reads, as opposed to 3.4% in WR. In contrast, *Nitrospirota* represented an average of 2.3% of the total reads in WM compared with 1.8% in WR.

**Figure 2:**
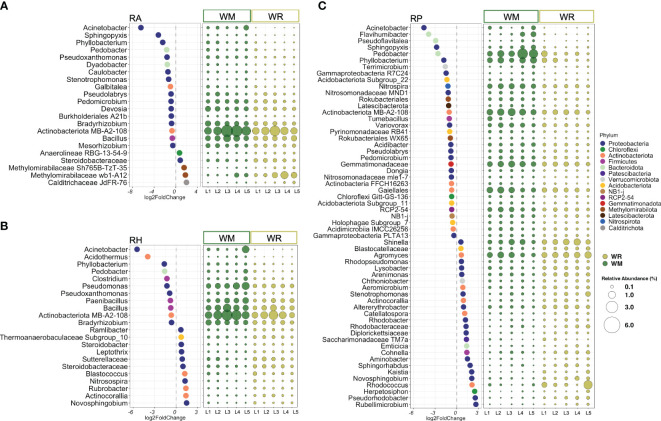
The most differentially abundant taxa between crop rotational positions, wheat monoculture (WM), and wheat rotation (WR) in **(A)** root-affected soil (RA); **(B)** rhizosphere (RH); and **(C)** rhizoplane (RP). Only taxa presenting an average abundance higher than 0.1% were displayed. The lowest confident classifications are shown, and the corresponding phylum presents a unique color. Negative log2FoldCange values indicated a significantly higher abundance in WM, while positive log2FoldChange values indicated a higher abundance in WR. The relative abundance of each significantly different taxa is represented by round shapes, ranging in size from 0.1% to 6% of the total sequences. *P* values < 0.05 followed by the Benjamini-Hochberg correction.

Because the microhabitat was the most influential factor in shaping the microbial communities, WM and WR were further analyzed separately by RA, RH, and RP. Taxa representing 13 phyla and two candidate phyla were found to be significantly different between WM and WR, with an average relative abundance higher than 0.1% of the total reads. In root-affected soil (RA), seven phyla were represented by 22 taxa, most of them belonging to the phylum *Proteobacteria*. From these, 17 taxa were statistically more abundant in WM, while only five were significantly more abundant in WR (**Figure 2A**). In RH, 22 taxa representing five phyla, *Actinobacteriota*, *Acidobacteriota*, *Bacteroidota*, *Firmicutes*, and *Proteobacteria*, were found to be significantly different in WM and WR (**Figure 2B**). Significant differential abundance was equally distributed between WM (n=11) and WR (n=11). Among the microhabitats, RP exhibited the highest number of significantly different taxa, which confirms the importance of this microhabitat in differentiating crop rotations (**Figure 2C**). Within the RP microhabitat, 12 phyla and two candidate phyla were represented by 59 taxa that exhibited differential abundance between the WM and WR samples. Among these taxa, 33 displayed significantly higher relative abundance in WM, whereas 26 showed a higher relative abundance in WR. Interestingly, taxa such as *Acinetobacter*, *Pedobacter*, *Phyllobacterium*, and the actinomycetes belonging to the candidate class MB-A2-108 were significantly more abundant in WM across all microhabitats. For instance, *Acinetobacter* exhibited a remarkable increase in abundance in WM, ranging from 4.5 to 6.5 times more abundant than WR in RP and RA, respectively.

Distinct patterns between WM and WR were noted when considering only taxa with high relative abundance (defined here as higher than 1% of the total reads) in at least one microhabitat. In RA, *Bacillus*, *Bradyrhizobium*, *Devosia*, and taxon MB-A2-108 exhibited higher relative abundances in WM than in WR (*p* = 0.014, *p* = 1.5e-05, *p* = 0.003 and *p* = 0.008, respectively) (**Figure 2A**). In RH, *Bacillus*, *Bradyrhizobium*, and the taxon associated with the actinomycete candidate class MB-A2-108 also displayed a significantly higher abundance in WM relative to WR (*p* = 0.021, *p* = 8.4e-05, and *p* = 0.021, respectively) along with *Paenibacillus* and *Pseudomonas* (*p* = 0.024 and *p* = 0.004, respectively) (**Figure 2B**). Within RP, six taxa, including *Nitrospira*, *Pedobacter*, *Phyllobacterium*, taxa within the order *Gaiellales* and class MB-A2-108 (phylum *Actinobacteriota*), along with a taxon from the family *Gemmatimonadaceae*, exhibited significantly higher relative abundances in WM compared to WR (*p* = 9.8e-09, *p* = 2.1e-05, *p* = 0.0003, *p* = 0.002, *p* = 1.7e-10, and *p* = 1.7e-06, respectively) (**Figure 2C**). Only two genera, *Agromyces* and *Shinella*, displayed significantly higher abundances in WR than in WM (*p* = 0.016 and *p* = 0.048, respectively).

### 3.3 Microbial diversity and composition in the rhizoplane

**Figure 3:**
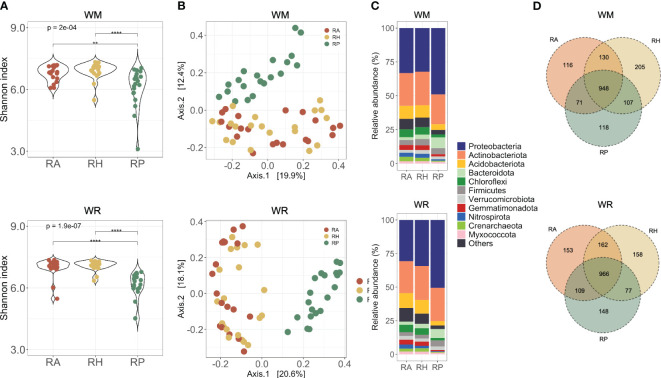
Microbial communities collected in different microhabitats (RA = root-affected soil; RH = rhizosphere; RP = rhizoplane) under WM, wheat monoculture; and WR, wheat rotation. **(A)** Alpha diversity of microbial communities based on the Shannon diversity index; **(B)** Beta diversity of microbial communities based on Principal Coordinates Analysis using Bray-Curtis dissimilarity values; **(C)** Most abundant taxa presented in the samples; Shapes indicate the significant differences between microhabitats: triangle, significant differences between RA and RH; circle, significant differences between RA and RP; square, significant differences between RH and RP; **(D)** Shared taxa in different soil depths for wheat rotational positions. Statistical differences across microhabitats are indicated by asterisks (** *p* < 0.01; **** *p* < 0.0001).

The rhizoplane harbored significantly less diversity than the other microhabitats studied, independent from the crop rotation scenarios evaluated (WM, *p* = 2e-04; WR, *p* = 1.9e-07) (**Figure 3A**). Dissimilarities in microbial communities using multidimensional scaling (MDS) plots based on Bray-Curtis distances (**Figure 3B**) showed significant distinctions between RP and the other microhabitats (PERMANOVA, *p* < 0.001). These differences underscore the influence of microhabitats on the microbial communities, which contributed to 8-15.8% of the observed variation in both crop rotational scenarios (**Supplementary Table 3**).

Distinct microbial compositions in the rhizoplane of WM and WR compared with the other microhabitats were observed primarily at the phylum level, based on the relative abundance of the most abundant phyla (**Figure 3C**; **Supplementary Figure 2**). Eight phyla exhibited significant abundance in WM RP compared with RA and RH. Specifically, *Proteobacteria* and *Bacteroidota* presented an increased relative abundance in RP (*p* = 0.001) (**Supplementary Figure 2**). Conversely, *Acidobacteriota*, *Chloroflexi*, *Crenarchaeota* (*Thaumarchaeota*), *Gemmatimonadota*, *Myxococcota*, and *Nitrospirota* exhibited a decrease in the relative abundance in RP compared to the other microhabitats (*p* < 0.001). Similar trends were observed in WR, with the addition of *Firmicutes* to the list of phyla that were significantly more abundant in RP than in other microhabitats (**Supplementary Figure 2B**). Interestingly, *Bacteroidota* and *Nitrospirota* presented an oppositely prominent gradient toward the roots. *Bacteroidota* increased significantly towards the roots in WM (*p* < 0.003), whereas *Nitrospirota* decreased consistently in relative abundance near the roots in WR (*p* < 0.014).

The microhabitat taxa composition showed 1,695 taxa in the WM samples and 1,773 in the WR samples (**Supplementary Table 1**). The core comprised 948 taxa in WM and 966 taxa in WR, indicating that more than half of the taxa (average 55.5%) were found in all three microhabitats (**Figure 3D**). More taxa were shared between microhabitats RA and RH than between RA and RP or RH and RP. Independent of the cropping history, unique taxa in each microhabitat were present only at low relative abundances (up to 0.02% of the total sequences).

As anticipated by the overall analyses, our method of separating the microorganisms from the rhizoplane was efficient. *Agromyces*, *Bradyrhizobium*, *Brevundimonas*, *Devosia*, *Pedobacter*, *Pseudomonas*, *Shinella*, *Sphingomonas*, *Sphingopyxis*, *Streptomyces*, and *Variovorax*, were the genera significantly more abundant in the RP of WM than in RH and RA (**Supplementary Figure 3A**). In WR, all the genera cited above and *Paenibacillus* were found in significantly higher relative abundance in RP (**Supplementary Figure 3B**). Looking specifically at the taxa in RP, *Bradyrhizobium*, *Nitrospira*, *Pedobacter*, and *Sphingopyxis* were significantly more abundant in WM, while *Agromyces* and *Shinella* were significantly more abundant in WR (**Supplementary Figure 4**).

### 3.4 Microbial composition over soil depth and microhabitats between crop rotational positions

**Figure 4:**
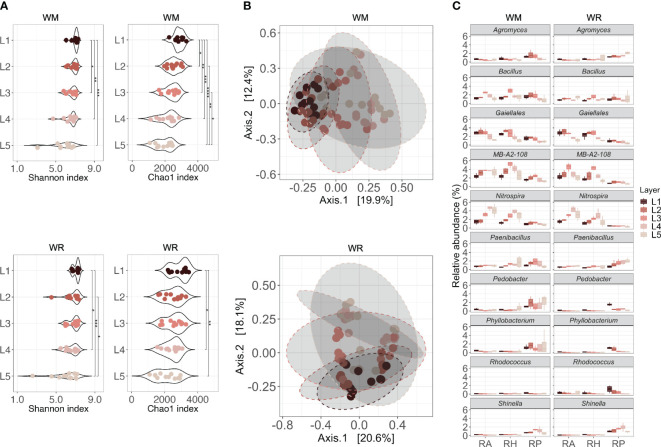
Microbial communities collected in different soil depths (L1 = 0-15 cm; L2 = 15-30 cm; L3 = 30-60 cm; L4 = 60-90 cm; L5 = 90-120 cm) under WR, wheat rotation; and WM, wheat monoculture. **(A)** Alpha diversity of microbial communities based on the Shannon diversity index and Chao1 index; **(B)** Beta diversity of microbial communities based on Principal Coordinates Analysis using Bray-Curtis dissimilarity values; **(C)** Shared taxa in different soil depths for both wheat rotational positions. Statistical differences in different microhabitats across soil depths are indicated by asterisks (* *p* < 0.05; ** *p* < 0.01; *** *p* < 0.001; **** *p* < 0.0001).

A link between soil depth and microbial community alpha diversity was observed in both wheat rotational positions (**Figure 4A**). There was a significant difference in microbial diversity across soil depths (WM, *p* < 0.001; WR, *p* < 0.01). The deeper the layer, the lower the diversity and richness, which decreased from L1 to L5. L1 and L2 harbored significantly higher microbial diversity than the other layers, but richness was already lower in L2 compared to L1. Interestingly, L1 and L2, which represent the first 30 cm (topsoil), were not significantly different from each other in WR (*p* ≥ 0.05) but were significantly different in WM (*p* < 0.05). The beta diversity, measured as the average Bray-Curtis dissimilarity between samples, also showed that approximately 21% of the differences in the WM community structure could be explained by soil depth, and 18.2% in WR (PERMANOVA, *p* < 0.001) (**Figure 4B**).

In the RA and RH of WM, *Bacillus*, *Nitrospira*, and MB-A2-108 had a higher relative abundance in the first layers and declined after L3 or L4 (*p* < 0.05; **Figure 4C**). A similar trend was observed for a taxon in the order *Gaiellales*, with high relative abundance in the first layers and a decline after L3 (RA, *p* = 0.0226; RH, *p* = 0.0081). The same trend was observed in WR, except for *Bacillus*, which showed no statistical difference between layers (*p* > 0.05). In RP, *Agromyces* declined significantly over depth in the rhizoplane of WM (*p* = 0.020). *Shinella* presented a similar pattern, increasing in L3 and reducing after that (*p* = 0.037). In WR, *Paenibacillus* increased over depth (*p* = 0.046), whereas *Pedobacter*, *Phyllobacterium*, and *Rhodococcus* declined after L1 or L2 (*p* = 0.018, *p* = 0.0055 and *p* = 0.024, respectively).

## 4 Discussion

Numerous studies have emphasized the positive impacts of rotational cropping systems, including improvements in soil fertility, plant health, and crop yields (Babin et al., 2019; Cheng et al., 2023; Sun et al., 2023; Town et al., 2023). In the present study, we explored the influence of crop rotational practices, specifically wheat in rotation vs. wheat monoculture, on the diversity of top- and subsoil microorganisms across three distinct soil- and root-associated microhabitats and five soil depths. Our study reveals that the rhizoplane of winter wheat specifically hosts a diverse community of microorganisms, and these communities exhibit some adaptability based on crop rotation. Furthermore, our findings demonstrate that the microbial communities colonize the entire root system, reaching depths of at least 120 cm. These microorganisms seem recruited by the plant (root exudates as a nutrient source) and allocated to the rhizoplane. Some of them may harbor genes related to nitrogen cycling. This recruitment potentially fulfills the plant’s nutritional requirements across its entire root system.

### 4.1 Monoculture impacts the abundance, diversity, and composition of microbial communities

Monoculture is commonly linked to a decline in agricultural productivity and yield performance over time compared with systems that use diversified crop species in rotation (Smith et al., 2023). Arnhold et al. (2023a) studied the effects of crop rotational position on wheat biomass in the same field plots in Harste. They found, on average, 54% higher aboveground biomass in wheat following winter oilseed rape compared with wheat monoculture in early plant stage BBCH 30, confirming earlier studies by Sieling et al. (2005); Sieling et al. (2007). They also demonstrated a significantly higher root length density after oilseed rape as a pre-crop than wheat as a pre-crop in the subsoil at BBCH 69 (Arnhold et al., 2023b). These results correlated well with wheat biomass formation, and differences in wheat aboveground biomass that occurred at early growth stages in spring remained persistent until harvest (Arnhold et al., 2023b).

We hypothesized that the microbiota associated with wheat under different crop rotational positions would exhibit variations in abundance, diversity, and composition. Our investigation into the absolute abundance of 16S rRNA gene copies revealed a disparity: in 60-120 cm deep (L4 and L5), WM presented an approximately threefold higher increase in 16S rRNA gene copies compared to WR, suggesting that crop rotation somehow impacts the root-associated microbial communities in the deeper soil layers. *Phyllobacterium* and *Pedobacter* were the bacterial genera with higher relative abundance in L4 and L5 in WM compared to WR. In addition, *Pedobacter* was also among the top five bacterial genera isolated from the rhizoplane by culturomics in the same soil (data not shown). As Hansen et al. (2020) highlighted, a similar phenomenon was observed when camelina (an oilseed crop belonging to the family *Brassicaceae*, as is oilseed rape) was introduced into a monoculture wheat system. They observed a decline in the abundance of microbial groups, such as fungi, mycorrhizae, gram-positive and gram-negative bacteria, and total microbial abundance in crop rotational samples. Such an occurrence may be attributed to introducing a novel crop into an established monoculture, which can disrupt the stability of the soil microbial communities. However, the exact reason for the significantly higher bacterial 16S rRNA gene copies in WM compared with WR in L4 and L5, especially considering the stable root biomass after L2, remains unclear. We speculate that the difference in the abundance of microorganisms might be due to the distribution pattern of wheat roots. The effective root zone - where most crop roots are concentrated - is typically estimated to be within the range of approximately the top 100 cm in depth (Fan et al., 2016), where we found more 16S rRNA gene copies. However, our sampling methodology involved taking a pivotal core (6 cm diameter) from the core wheat plant rather than sampling the entire plant, which could explain the lower root biomass obtained. In other words, we might have dealt with a sampling artifact because only four cores for each crop system were sampled.

The relative abundance of bacterial N-cycle-related genes in the rhizoplane (closest proximity to the root), including *nifH*, *nosZ*, and *amoA* genes, was assessed to explore potential correlations with taxa engaged in N-cycling, such as belonging to the phylum *Nitrospirota*, predominantly found in the deeper soil layers (subsoil). We inferred that certain bacterial taxa play a significant role in nitrogen acquisition by plants. However, the gene copy numbers associated with N-cycling in the rhizoplane samples exhibited variations across different soil depths, and no disparities were observed between WM and WR. Liu et al. (2023) noted that crop rotation is a significant factor influencing nitrogen-cycling functional genes. They found that crop rotation increased the richness of bacterial communities associated with functional genes involved in nitrogen cycling.

A higher relative abundance of the ammonia monooxygenase gene *amoA* correlates well with a slightly higher relative abundance of known nitrifying bacteria such as *Nitrospira* (Daims et al., 2015) in L1 of WM. In addition, *Pedobacter* was found to have higher relative abundance in 60-120 cm deep (L4 and L5) of WM. This observation might suggest two possibilities: either an enhanced capacity of wheat roots for nitrogen acquisition or a necessity for additional nitrogen to stimulate plant growth, potentially compensating for root biomass loss. Some *Pedobacter* species have been identified in nitrifying inocula (Vanparys et al., 2005), although their exact role as nitrifying bacteria remains not fully understood. Isolates classified as *Pedobacter* from the same location in the same year displayed cellulase, glucanase, protease, and phosphatase activity as well as phytohormone (ACC-deaminase and IAA) production and antifungal capabilities (data not shown), most likely contributing to plant growth promoting effects.

Our findings revealed that wheat microbiomes associated with wheat crop rotation and wheat monoculture exhibit comparable alpha diversity indices. This observation does not align with existing research in the field. For example, a diversified cropping system has shown positive effects on the soil microbiome, which can be observed by the increase in alpha diversity and expanded network structure in soils under conventional N application (Liu et al., 2023). A meta-analysis conducted by Venter et al. (2016) found that higher microbial diversity was generally observed in soils with a greater diversity of crops. They suggested that this relationship might become less pronounced in longer-term field trials. In the context of our research, it is crucial to highlight that the samples collected for the WR originated from a mix of wheat plots following either oilseed rape or wheat cultivation. Therefore, it is plausible that our findings are influenced by various effects stemming from the overall diversity of the crop rotation and the specific pre-crop (i.e., oilseed rape or wheat), as also indicated by Hilton et al. (2018). Notably, a study investigating core soil bacterial genera and enzyme activity in soils from century-old wheat rotations in the Canadian prairies reported a decreased alpha diversity of soil bacteria in wheat monoculture (Lupwayi et al., 2021a). Peralta et al. (2018) found a 4% lower bacterial diversity in a diversified crop rotation (corn-soybean-wheat with two cover crops) than in a maize monoculture. This finding contrasts with the absence of a significant effect observed in our study and the positive correlation reported by Venter et al. (2016).

Indeed, alpha diversity alone may not yield a sufficient understanding of the differences observed in these communities. As Shade (2017) emphasized, it is crucial to consider within-sample diversity as a foundational aspect when identifying components and developing hypotheses to comprehend the ecological mechanisms at play. Our findings indicate that the differences in monoculture relative to crop rotations may be related to the selective microbial recruitment by the root system (Ling et al., 2022). Even at the higher taxonomic level of the phylum, we observed a notable increase in the abundance of *Firmicutes* and *Nitrospirota* within the monoculture context. These findings align with those of Lupwayi et al. (2021b), where *Firmicutes*, particularly the class *Bacilli*, exhibited a higher relative abundance in wheat monoculture than in wheat-pea cropping systems. It is worth noting that both *Firmicutes* and *Nitrospirota* are recognized as copiotrophic bacteria, characterized by their rapid growth and active involvement in nitrogen cycling processes.

In a more detailed examination, differential analyses have highlighted significant distinctions within the microbial community between the two cropping systems, WM and WR, across all microhabitats assessed at the genus level. Within these distinctions, many well-known putative plant growth-promoting bacteria (for example, *Bacillus*, *Bradyrhizobium*, and *Pseudomonas*) exhibited higher relative abundance in WM. In contrast, a few genera, including *Agromyces* and *Shinella*, previously associated with wheat or rapeseed microbiomes, exhibited increased abundance in WR. These genera are known to possess capabilities related to nitrogen fixation and nitrate reduction (Jimenez-Gomez et al., 2020; Taye et al., 2020; Kavamura et al., 2021). This observed pattern may indicate the plant’s strategy to recruit potentially beneficial microorganisms to cope with the effects of monoculture or address potential nutrient leaching resulting from monoculture practices.

### 4.2 Microbial responses in different microhabitats

Although the microbial diversity indices were similar between WM and WR, there were notable differences in how microbial communities were structured across various microhabitats. Our observations showed that RA and RH displayed greater diversity and richness than RP in both crop rotational positions. The plant-soil continuum, known for its intricate and diverse nature and significant spatial variability, plays a key role in shaping the microbial community composition in the soil (Berg & Smalla, 2009). The rhizoplane is typically considered the hub of soil microbial activity due to its rich nutrient content. Our data corroborate with those of Xiong et al. (2021), who highlighted the decrease in diversity and richness from bulk soil and rhizosphere compared with the rhizoplane of maize, wheat, and barley from 468 samples collected in China. Other methodologies, such as sonication, have also efficiently separated these microhabitat communities and yielded similar results in wheat and rice (Edwards et al., 2015; Richter-Heitmann et al., 2016: Attia et al., 2021).

As observed in studies involving various species of plants, there is a decline in microbial diversity and complexity as one moves from the broader soil environment (e.g., bulk soil, root-affected soil, rhizosphere) toward the more direct proximity to the plant (e.g., rhizoplane and phyllosphere) (Xiong et al., 2021). The difference in the shared taxa between RA and RP can provide insights into the dynamics of this process. RA and RP in WM shared fewer taxa than the RH and RP soil (71 and 107 taxa, respectively). However, in the case of WR, the RA and RP shared more taxa than the RH and RP soil (109 and 77 taxa, respectively). This change in the shared taxa between the microhabitats may be attributed to the specific characteristics of the different rotational positions and alterations in the physicochemical soil properties due to tillage. It is well-established that the choice of the preceding crop has a significant impact on the microbial community of the subsequent crop (Hilton et al., 2018; Babin et al., 2019), and different microbial rearrangements may occur each time the crop changes. In the case of monoculture, the root-affected soil already harbors the necessary microbes for the plant (same plant residues left on the field each year), leading to different results in the shared taxa between microhabitats compared to rotation.

We efficiently differentiated RH and RP using a combination of approaches (Lucas et al., 2018). Upon analyzing microorganisms commonly associated with the wheat rhizosphere (Chen et al., 2019; Chen et al., 2021b; Elvia et al., 2021; Kavamura et al., 2021; Prudence et al., 2021), we observed that certain microorganisms were indeed highly abundant on the rhizoplane. It is likely that the greater abundance of *Proteobacteria* in the rhizoplane is due to increased root accessibility to nutrients (Ho et al., 2017; Ling et al., 2022). For example, taxa from the phyla *Proteobacteria* (e.g., *Devosia*, *Pseudomonas*, and *Shinella*), *Bacteroidota* (e.g., *Pedobacter*), and *Actinobacteriota* (e.g., *Streptomyces*) were found to increase in the RP significantly. The study conducted by Balbin-Suarez et al. (2020) investigated the effects of different substrates on *Apple Replant Disease* (ARD). They highlighted the importance of studying rhizoplane and rhizosphere separately to discern microbial communities associated with healthy and diseased states. In our study, a specialized and efficient strategy for separating microhabitats was used to catch the flow of specific taxa of bacteria residing in root-affected soil and rhizosphere and allocate themselves close to the plant in the rhizoplane. Although the endosphere has not been evaluated, it is also promising in its ability to host microbiota specific to different cropping systems, such as those observed in other microhabitats.

### 4.3 Microbiome response to soil depth

Little is known about the microbial communities in deeper soil layers associated with the roots of crops grown in rotation compared with monoculture. A few studies have performed microbial diversity analyses in wheat soil layers deeper than 30 cm (Uksa et al., 2017; Schlatter et al., 2018; Schlatter et al., 2020a; Schlatter et al., 2020b), but these studies focused on something other than cropping histories or detailed rhizo-compartmentalized microbial communities. In this study, we systematically addressed the microbial communities in different microhabitats near roots (from the surrounding soil, rhizospheric soil, and root surface) of winter wheat grown in rotational and monoculture field plots in soil layers up to 120 cm deep. After the first 15 cm depth, root biomass decreased drastically in both field plots.

Wheat roots concentrate their biomass in the uppermost soil layers, particularly within the 0-20 cm range (Fan et al., 2016). For instance, in the case of wheat, an estimated 95% of its root biomass can be found in the top 100 cm of soil and 50% in the top 0-20 cm (Thorup-Kristensen et al., 2009; Fan et al., 2016). This upper soil layer is typically a microbial hotspot, boasting higher diversity and abundance. This microbial increase is attributed to the availability of crucial resources such as oxygen, nutrients, water, and organic matter (Hao et al., 2021). Our findings indicate that the microbial community significantly decreased over depth. This divergence is likely due to the dynamic nature of the topsoil community and its heightened responsiveness to environmental fluctuations, such as temperature, pH, and moisture variations, compared with the subsoil microbial community. However, it is important to note that deep-rooted plants can also significantly influence soil development processes and carbon storage (Maeght et al., 2013; Leewis et al., 2022). These patterns in the root distribution over depth may be attributed to the soil’s physical structure from both monoculture and rotational wheat. However, in the study conducted by Arnhold et al. (2023a) in Harste over two years, no differences in soil structural parameters (i.e., bulk density, total pore volume, air and field capacity, and aggregate stability) were found for an early sampling in April (BBCH 30) over three soil depths under wheat grown in different crop rotational positions.

In the deeper soil layers, the associated microbial communities can exhibit considerable diversity or richness (Steger et al., 2019), especially under a wetter climate (Leewis et al., 2022). In our study, despite these disparities, diversity and richness remained notably high even in the deepest layer. Regarding composition, except for a few taxa with low relative abundance, all were found at different soil depths, indicating a structured microbial organization from topsoil to deep soil.

Except for a few taxa with low relative abundance, all were found at different soil depths, indicating a structured microbial organization from topsoil to deep soil. However, there was a more prominent fluctuation in the relative abundance of *Bacillus*, *Nitrospira*, and actinobacteria taxa (MB-A2-108 and *Gaiellales*) from top- to subsoil layers in either RA, RH, or both, with a gradient, that exhibited a trend of increasing relative abundance extending to a depth of 60 cm. *Nitrospira* plays a crucial role in nitrification, which involves the conversion of nitrite to nitrate. This process requires an appropriate balance of oxygen and ammonium, which can vary across different soil depths. Topsoil layers, characterized by elevated organic matter content and higher microbial activity, are likely to provide a more plentiful supply of ammonium and oxygen, thus fostering the growth and metabolic activity of ammonia oxidizer archaea (AOA) and bacteria (AOB), such as those from the family *Nitrosphaeraceae*, also found in high abundance in RA in our study. Similar findings regarding the high relative abundance of *Nitrospirae* (*Nitrospirota*) and MB-A2-108 (*Actinobacteriota*) were reported by Steger et al. (2019) in their investigation of floodplain soil bacterial composition across various soil depths, ranging up to 2 meters, except the surface soil. The authors also noted an exponential decrease in soil organic carbon and nitrogen concentrations with increasing soil depth.

Shifts in the relative abundance of various microorganisms were evident across distinct soil depths, with several strains within these genera previously identified as putative plant growth-promoting rhizobacteria (PGPR), intrinsically associated with the microbiomes of wheat and rapeseed. Many of these strains are recognized for their roles in the nitrogen cycle. Notably, nitrite-oxidizing bacteria (*Nitrospira*) and diazotrophs (*Agromyces*, *Paenibacillus*, *Phyllobacterium*, *Rhodococcus*, and *Shinella*) displayed a positive correlation with higher levels of nitrogen fertilization, consistent with the findings reported by Chen et al. (2021b). In our study, conducted during the flowering stage under conditions of elevated nitrogen fertilization, we observed a consistent presence of potential PGPR. This suggests that plants may utilize the secretion of organic acids as an adaptive strategy to attract beneficial microorganisms in response to elevated nitrogen inputs in intensive agricultural systems (Chen et al., 2019). However, our assessment of bacterial N-cycle-related genes in the rhizoplane, including *nifH*, *nosZ*, and *amoA*, showed no significant differences between the WM and WR conditions. This implies that the microbial functional potential associated with nitrogen cycling remained relatively stable across the two crop rotational positions and soil depths. The observed decline in bacterial *amoA* with increasing depth, coupled with the rise in *nifH* is not fully in agreement with the literature. Liu et al. (2022) conducted a study comparing functional gene abundance related to nitrogen between topsoil and subsoil. Their findings indicated a decrease in the abundance of nitrogen functional genes (*nifH*, *amoA*, *nirK*, *nirS*, and *nosZ*) in the subsoil (30-40 cm) when compared to the topsoil (0-10 cm) in grasslands. However, some variation occurred depending on the grassland habitats. In another study, the abundance of functional N-cycle genes decreased with depth for agricultural soils cultivated with maize (Zilio et al., 2020). The N-cycle gene copies abundance was higher in the surface layers (0–50 cm) than in deeper layers (50–100 cm).

In monoculture systems, cultivating the same crop repetitively in the same field year after year can lead to a detrimental decline in soil health, occasionally resulting in yield decline. This continuous exposure to uniform environmental conditions may also result in soil microbiome dysbiosis, as highlighted by Wu et al. (2018); Wu et al. (2020). The intricate process of microbial transmission from the surface to deeper soil layers through plant roots is influenced by various factors, including the movement of water, nutrients, and organic matter, secretion of root exudates and mucilage, and formation of physical root channels. Our comprehensive sampling approach, involving deep soil cores, allowed us to uncover variations in the abundance of specific taxa associated with winter wheat. Remarkably, we identified the presence of putative PGPR along the entire extent of the wheat roots to depths of up to 120 cm. Like their counterparts in the topsoil layers, these bacteria may be vital in sustaining the plant’s health and the ability to thrive in subsoil layers characterized by lower organic matter and nutrient content. As we plan for future research focusing on root-associated microbiomes, it is essential to consider the contrast between the rhizoplane and the rhizosphere and explore microbiomes at various soil depths. Combined with investigations based on 16S rRNA gene amplification and quantification of genes of interest, techniques centered on microbial cultivation to isolate PGPR can also provide more insights regarding the microbiome’s relationship with soil depth and its impact on plant health. This information could lead to developing a sustainable farming approach for enhancing food security and ecosystem health, especially in upcoming climate scenarios where water scarcity may limit plant growth.

## Data availability statement

The datasets presented in this study can be found in online repositories. The names of the repository/repositories and accession number(s) can be found below: NCBI - PRJNA940322.

## Author contributions

AG: Conceptualization, Data curation, Formal analysis, Investigation, Methodology, Software, Validation, Visualization, Writing – original draft, Writing – review & editing. JA: Methodology, Writing – review & editing. DG: Methodology, Writing – review & editing. KS: Funding acquisition, Project administration, Resources, Supervision, Writing – review & editing. AB-K: Conceptualization, Data curation, Investigation, Methodology, Validation, Writing – original draft, Writing – review & editing.
