# Supplementary material for: Soil depths and microhabitats shape soil and root-associated bacterial and archaeal communities more than crop rotation in wheat
Source: Front Microbiomes. 2024 Feb 5;3:1335791. doi: 10.3389/frmbi.2024.1335791 (PMC12993652; doi:10.3389/frmbi.2024.1335791)
Supplement: Supplementary file 1 [file DataSheet_1.docx]

Supplementary Material

Soil depths and microhabitats shape soil and root-associated bacterial and archaeal communities more than crop rotation in wheat

**Adriana Giongo*, Jessica Arnhold, Dennis Grunwald, Kornelia Smalla, Andrea Braun-Kiewnick**

*** Correspondence:** Corresponding Author: [adriana.giongo@julius-kuehn.de](mailto:adriana.giongo@julius-kuehn.de)

# Supplementary Figures and Tables

## Supplementary Figures


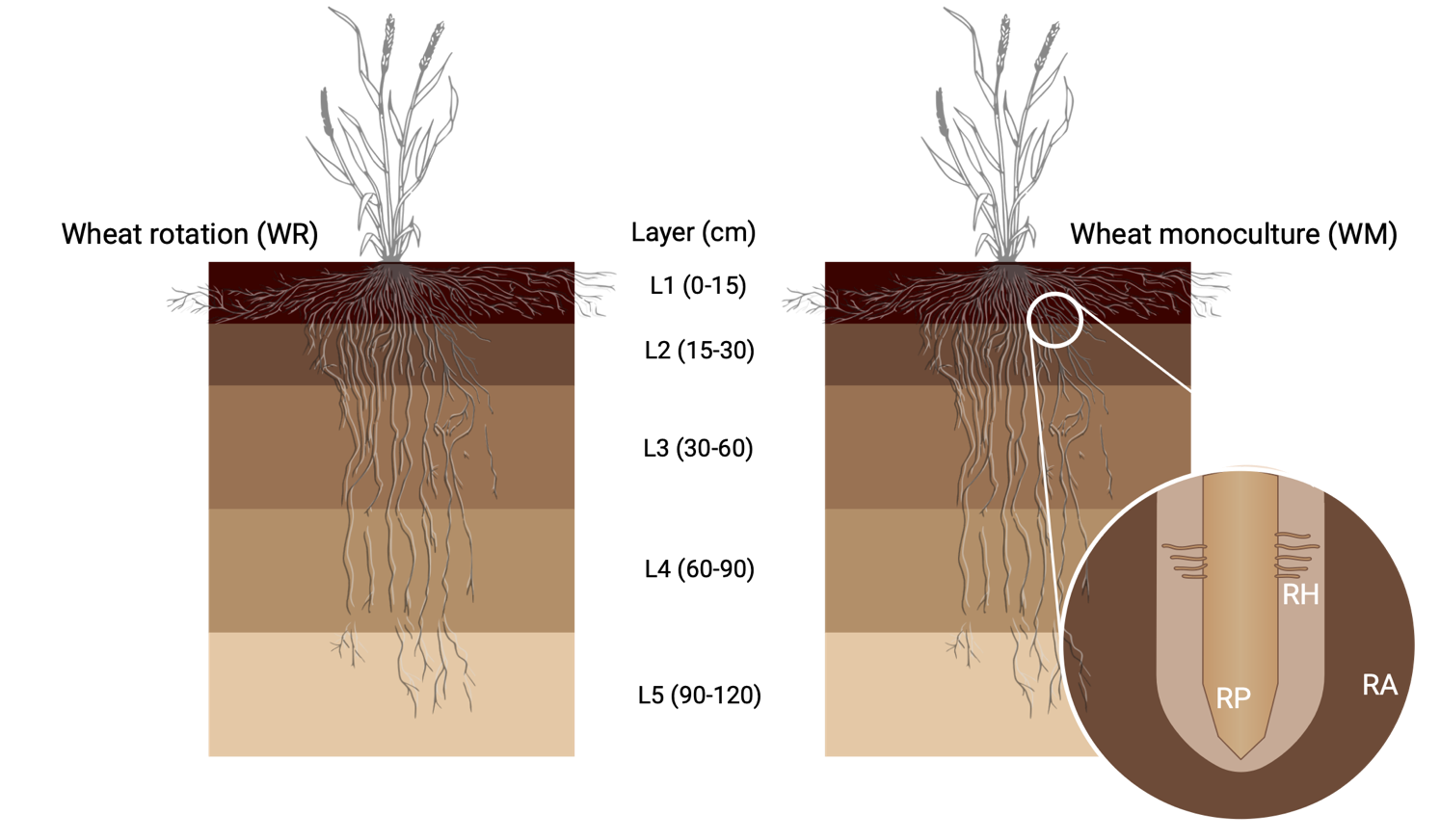


**Supplementary Figure 1.** Overview of the sampling procedure. Samples from three microhabitats (RA = root-affected soil; RH = rhizosphere; and RP = rhizoplane) were collected from wheat crop rotation (WR) and wheat monoculture (WM) fields at five soil depths. The figure was created using BioRender.com.


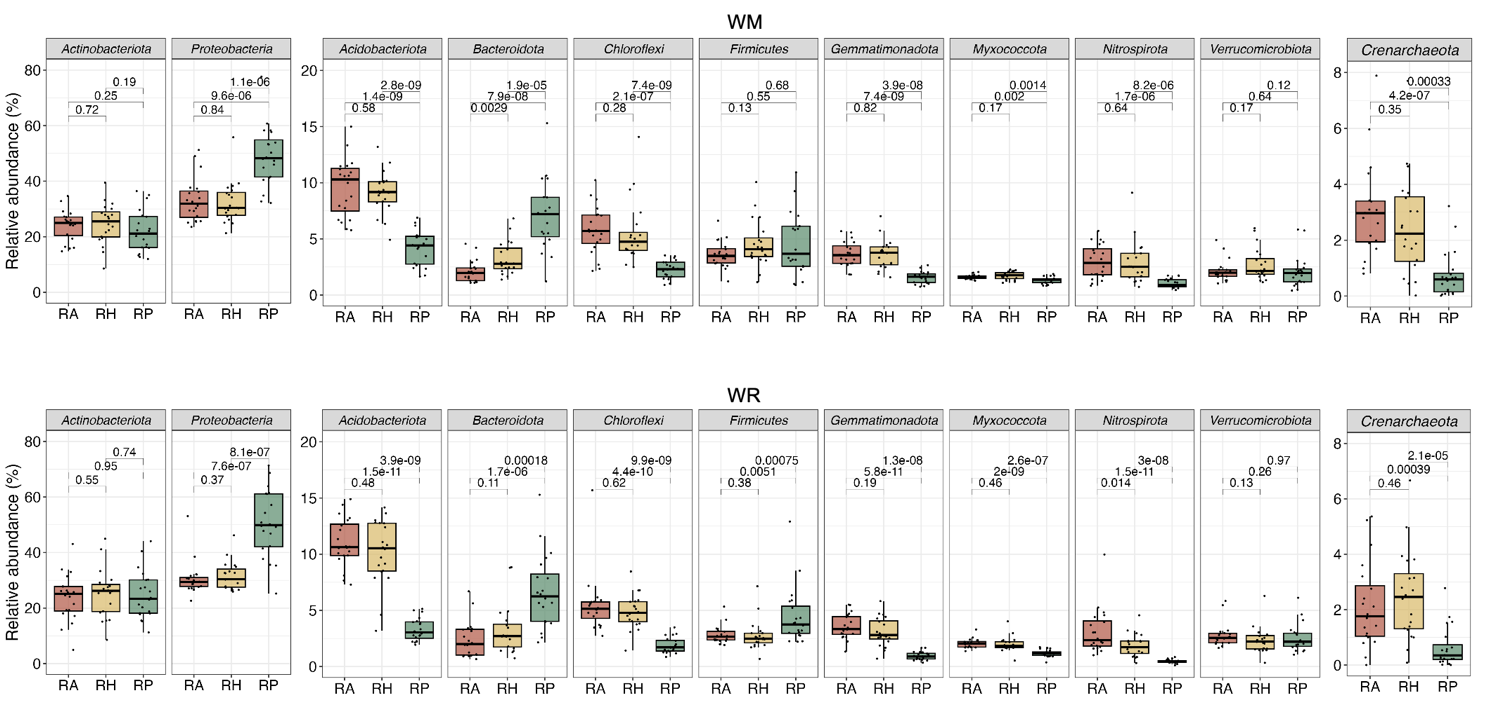


**Supplementary Figure 2.** Relative abundance of the main phyla observed in microhabitats of wheat under WM, wheat monoculture; and WR, wheat rotation. RA = root-affected soil; RH = rhizosphere; and RP = rhizoplane. Bars represent standard error; statistical significance at *p* values < 0.05.


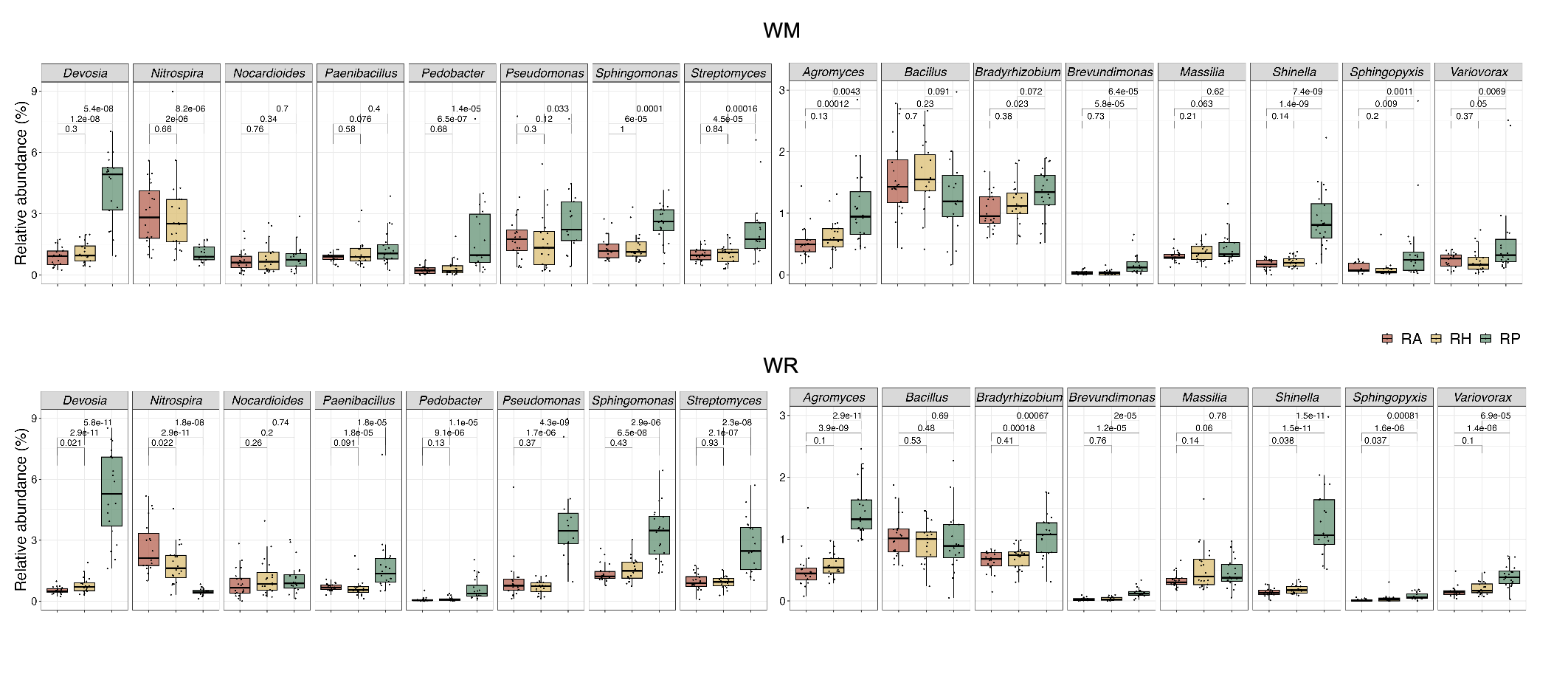


**Supplementary Figure 3.** Relative abundance of genera associated with wheat under (A) WM, wheat monoculture; and (B) WR, wheat rotation, separated by microhabitats (RA = root-affected soil; RH = rhizosphere; and RP = rhizoplane). Bars represent standard error and statistical significance *p* values < 0.05.


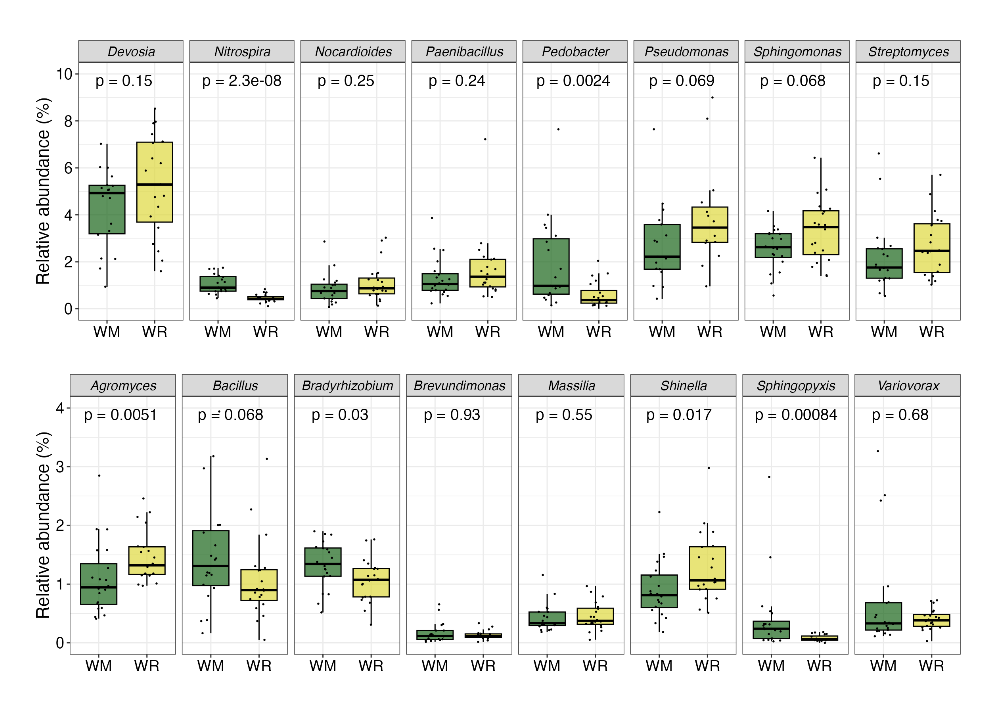


**Supplementary Figure 4.** Comparison of commonly associated root’s bacterial genera in crop rotational positions, wheat monoculture (WM), and wheat rotation (WR) in the rhizoplane of wheat. Bars represent standard error, and statistical significance *p* values < 0.05 between treatment means are displayed above bar plots.
